# Approach to the Patient with Achondroplasia—New Considerations for Diagnosis, Management, and Treatment

**DOI:** 10.1210/clinem/dgaf017

**Published:** 2025-01-15

**Authors:** Nadia Merchant, Julie Hoover-Fong, Ricki S Carroll

**Affiliations:** Division of Pediatric Endocrinology, Department of Pediatrics, University of Texas Southwestern Medical Center, Dallas, TX 75390, USA; Greenberg Center for Skeletal Dysplasias, Department of Genetic Medicine, Johns Hopkins University, Baltimore, MD 21218, USA; Division of Orthogenetics, Department of Pediatrics, Nemours Children’s Hospital, Delaware, Wilmington, DE 19803, USA

**Keywords:** achondroplasia, skeletal dysplasia, foramen magnum stenosis, vosoritide, CNP

## Abstract

Achondroplasia is the most common disproportionate short-stature skeletal dysplasia. Features associated with achondroplasia are rhizomelia, macrocephaly, midface hypoplasia, and typical cognition. Potential medical complications include foramen magnum stenosis, hydrocephalus, middle ear dysfunction, obstructive and central sleep apnea, spinal stenosis, and genu varum. Recently, vosoritide, a C-type natriuretic peptide analogue, was approved by the Food and Drug Administration with the primary indication of increasing linear growth in all children with achondroplasia and open growth plates. Due to this, pediatric endocrinologists suddenly are encountering infants and children with achondroplasia in their clinic whose families are seeking treatment with vosoritide. There is an urgent need to provide practical guidance pertaining to the diagnosis, management, and surveillance of these patients. Specific to current clinical use of vosoritide and other growth-modulating therapies in development for patients with achondroplasia, it is important to recognize that 1. some children and their families do not automatically desire such treatment, 2. not all treated children exhibit a response in linear growth, and 3. treatment does not negate the necessity of actively surveilling for the potential complications of achondroplasia that are part of its natural history. The goal of this paper is to provide probable, contemporary clinical scenarios of infants and children with achondroplasia who may present to an endocrinologist. This information is especially crucial to the endocrinologist when there is no specialized skeletal dysplasia center near the family.

Achondroplasia is an autosomal dominant genetic disorder caused by heterozygous gain-of-function variants of the fibroblast growth factor receptor 3 (*FGFR3*) gene with greater than 99% having a pathogenic variant in the same nucleotide regardless of ethnicity (ie, c.1138G > A or c.1138G > C; (p.Gly380Arg)). More than 80% of affected individuals have a de novo *FGFR3* variant and are born to average-stature parents (1). The incidence of achondroplasia is estimated to be 1 in 10 000 to 30 000 live births (2-4).

Characteristic clinical features of achondroplasia include short limbs with rhizomelia, macrocephaly, midface hypoplasia, and typical cognition (5-8). There are multiple potential medical complications occurring throughout the lifespan of a person with achondroplasia, requiring surveillance and management at age-appropriate times. Some of these complications are life-threatening, including foramen magnum (FM) stenosis, hydrocephalus, and sleep-disordered breathing. Other complications include middle ear dysfunction, spinal stenosis, and genu varum (Fig. 1).

**Figure 1.**
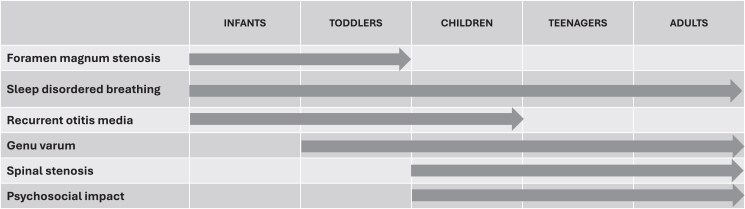
Complications at different stages of life in patients with achondroplasia.

For a child with achondroplasia, annual growth velocity (AGV) is slower than their average stature peers, averaging approximately 4 cm/year starting at school age (9-11). There is no appreciable pubertal growth spurt, and final adult height is typically 6 to 7 SDs below the general population mean. Recently vosoritide has been approved in multiple countries. In the United States, vosoritide was initially approved by the Food and Drug Administration (FDA) November 19, 2021, for those older than 5 years and the indication was then expanded Oct 20, 2023, to increase linear growth starting at birth (12, 13). Although vosoritide currently is the only drug approved by health commissions, there are several other investigational pharmaceuticals in development for the same indication.

With the approval of vosoritide, many families are presenting to their local endocrinologist to learn about and access this growth-modulating therapy. Importantly, if the patient is not followed in a specialized skeletal dysplasia center, he or she will require assessment, surveillance, and treatment for achondroplasia-related medical complications. There are health supervision guidelines and consensus statements published on medical management for achondroplasia by the international community, the American Academy of Pediatrics (AAP), and the European Achondroplasia Forum. There are also consensus guidelines for monitoring patients on vosoritide published by an international group of experts as well as a committee in Australia (1, 2, 8, 14-16).

Additionally, pediatric endocrinologists should be aware that vosoritide is the first treatment to increase linear growth in patients with achondroplasia, but several other investigational pharmaceuticals are in the developmental pipeline (7, 17). Therefore, treatment with this group of growth-modulating therapies will require periodic reassessment over time. Contrary to typical experience in the endocrine clinic with short-stature individuals with growth hormone deficiency, for example, children with achondroplasia and their families may seek information about the new therapy(ies), but opt not to treat. And most important, treatment with vosoritide and any other future growth modulation does not negate the necessity of actively surveilling for the potential complications of achondroplasia that are part of its natural history.

What follows are 4 clinical scenarios designed to illustrate age-specific achondroplasia-related issues that will arise in the process of considering treatment with vosoritide and other growth-modulating therapies. Pediatric endocrinologists should expect these situations will arise in their daily practice, today and in the coming years, as more pharmaceuticals are developed.

## Clinical Scenarios

### Take Home Points for Case on Prenatal Diagnosis of Achondroplasia

1. Signs of achondroplasia are not typically seen until the third trimester of pregnancy, at the earliest, and may not be obvious at birth by physical examination (18). After birth, a diagnosis of achondroplasia may be confirmed by a radiograph or genetic testing.
2. Expanded single-gene noninvasive prenatal testing (NIPT) during pregnancy is leading to earlier diagnosis (19, 20).
3. The FDA approval of vosoritide at birth paired with earlier NIPT diagnosis is leading to infants with achondroplasia presenting to endocrinologists in infancy and early childhood. It is imperative that endocrinologists be aware of achondroplasia health surveillance guidelines during infancy.

### Case 1: Prenatal Diagnosis

A 36-year-old G3P2 woman with no significant past medical history or complications during pregnancy decided to pursue comprehensive prenatal single-gene screening NIPT (19). The NIPT results at 18 weeks’ gestation detected the fetal *FGFR3* pathogenic variant c.1138G > A (p.Gly380Arg), consistent with achondroplasia. The 20-week anatomy scan was unremarkable. The patient was referred for genetic counseling and a maternal fetal medicine consultation. Prior to delivery, she was referred to genetics for a virtual visit through fetal medicine to discuss a delivery plan and postnatal care. During the visit, she was advised that as an average-stature mother, she may be able to have a vaginal delivery (18, 21). The couple was educated on potential medical complications of achondroplasia in infancy. In addition, the geneticist discussed the recommendation for postnatal confirmation of achondroplasia via radiological imaging and/or genetic testing. The geneticist recommended identifying a skeletal dysplasia center for coordination of care including longitudinal growth and development assessments, and an early sleep study and imaging to assess for FM stenosis (1) and associated neurologic abnormalities that may necessitate surgical cervicomedullary decompression. The geneticist also discussed the evolving medical treatment landscape for achondroplasia available to increase growth velocity starting at birth.

## Discussion of Prenatal Diagnosis

Since achondroplasia is caused by a de novo *FGFR3* variant in 80% of cases and the clinical features may be subtle, the diagnosis will likely not be suspected in average-height biological parent pregnancy (1). Achondroplasia is not evident in the 18- to 20-week anatomy scan (18, 22). By the third trimester, a fetus with achondroplasia may have decreased femur length, macrocephaly with frontal bossing, depressed nasal bridge, brachydactyly with trident hand, and a collar hoop sign (ie, abnormal connection to diaphysis with relative overgrowth of the periosteum) (18, 23, 24), but there is often no indication to perform this late imaging and therefore the differences are not seen. Even after birth, achondroplasia may not be obvious since midface hypoplasia may be mild and newborn length is within normal range of average stature infants (10, 18, 25).

Due to the later onset of features, chorionic villus sampling at or before 12 weeks’ gestation and amniocentesis from 16 weeks onward is not routinely performed for the indication of suspected achondroplasia (22). However, the American College of Obstetrics and Gynecology now recommends NIPT that can be performed as early as 9 to 10 weeks’ gestation for all pregnancies to assess for aneuploidies because the risk of detecting one of these is greater than the risk of miscarriage in the pregnancy via invasive prenatal testing methods (26). In some prenatal settings, a limited single-gene or panel for copy number variants and/or single genes is offered by NIPT (19, 20). As of late 2024, this prenatal panel of single genes includes the achondroplasia-specific *FGFR3* pathogenic variant c.1138G > A or c.1138G > C (p.Gly380Arg) (20, 23). Testing for *FGFR3* condition can only be conducted by NIPT if the pregnant parent does not have the same condition, since the test will not distinguish between maternal and fetal *FGFR3* variant (20). This is the case presented earlier, which is leading to earlier diagnosis and the presentation of expecting mothers and support partners to skeletal dysplasia centers and experts.

For a mother with achondroplasia, a cesarean delivery is necessary due to small pelvis and cephalopelvic disproportion (1). In a pregnant average-height woman with a fetus suspected to have achondroplasia, a trial of labor should be offered as long as there are no other obstetric risks, with counseling for possible need for cesarean delivery due to macrocephaly (1, 21).

Also, a prenatal appointment prior to delivery with a skeletal dysplasia expert, pediatrician, fetal center, geneticist, and/or pediatric endocrinologist is recommended if achondroplasia is suspected (18, 22). Anticipatory guidance is provided along with briefly discussing the importance of routine surveillance, the specialists involved in care during infancy, along with currently available up-to-date treatment options starting at birth, outcome data, and ongoing clinical trials (1, 22). A delivery plan should be made as well as a postnatal coordinated care plan with a multidisciplinary team (22).

Vosoritide was approved October 20, 2023, by the FDA for the indication to increase linear growth starting at birth after it was first approved November 19, 2021, for those older than 5 years (12, 13). This has led to an increase in interest by some families to start treatment as early as possible. Given this, pediatric endocrinologists are faced with needing to navigate routine surveillance guidelines for infants with achondroplasia along with safe prescribing practices in this age group. In addition, counseling should include a discussion regarding realistic expectations, noting that currently vosoritide has only been shown to increase height *z*-score from baseline (27) and has not been shown to change the chance of medical or orthopedic complications related to achondroplasia. Finally, it is recommended that one discuss other clinical trials for pharmacological agents in achondroplasia, so that expecting parents are informed and aware of their options.

### Take Home Points on Case in Newborn Nursery

1. There are several complications that can occur in the first few years of life, including FM stenosis, hydrocephalus, and apnea. An infant should be monitored closely for signs of these complications as well as to ensure growth and development are appropriate.
2. A medical leader of an infant's achondroplasia care should be appointed, ideally prior to discharge from the well-baby nursery, as is the case for infants with other complex medical needs (28).
3. Comprehensive evaluation with assessment of apnea, neurological examination, and imaging to determine the need for surgical intervention for FM stenosis is imperative and can be life-saving.

### Case 2: Newborn Nursery

**Figure 2.**
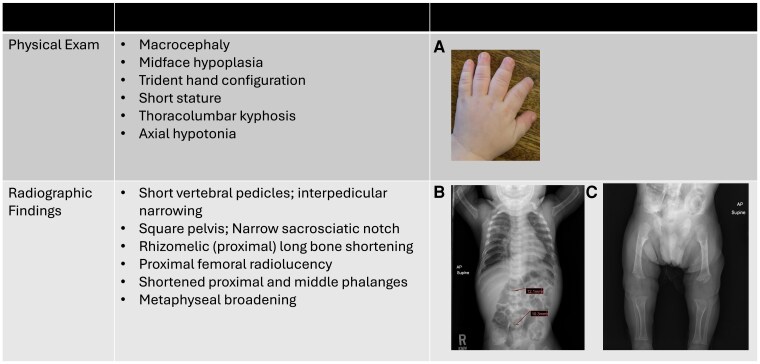
Features for clinical suspicion of achondroplasia after birth. A, Deviation between the third and fourth digits (“trident hands”). B, Decreased interpedicular distance from upper to lower lumbar spine. C, Flat rounded iliac bones with narrow sacrosciatic notches, and radiolucency of the proximal femurs. *Consent obtained from patient.*

A 1-day-old infant was evaluated in the newborn nursery. He was born at 39 weeks’ gestation to a 32-year-old healthy woman. The pregnancy was uncomplicated and the prenatal ultrasound at 20 weeks’ gestation was unremarkable. The infant was born via spontaneous vaginal delivery, and there were no clinical concerns following delivery. On examination, the infant was well-appearing, macrocephalic with frontal bossing, and had disproportionately short limbs as compared to the trunk with rhizomelia of the upper and lower extremities. The anterior fontanelle was soft, flat, and large at 5 cm by 4 cm. He had symmetric movement of all extremities, axial hypotonia, and normal appendicular tone with good strength and strong suck and grasp. The pediatrician suspected a skeletal dysplasia. A single radiograph including the infant's chest, abdomen, and extremities (eg, babygram) showed decreased interpedicular distance from upper to lower lumbar spine, flat rounded iliac bones with narrow sacrosciatic notches, and radiolucency of the proximal femurs, confirming the clinical diagnosis of achondroplasia (Fig. 2). Using achondroplasia male growth grids, length was 50%, weight was 50%, and head circumference was 25%. The newborn underwent a car seat test to screen for episodes of apnea and, given his achondroplasia diagnosis, particular attention to his lack of head control was noted. His pediatrician was made aware of the diagnosis and the family was scheduled for a visit with a local geneticist with a same-day baseline fast-spin magnetic resonance imaging (MRI) of the cervical spine and a coordinated overnight polysomnogram. The infant was discharged to home with his parents on day of life 2 in a car bed.

## Discussion with Newborn Nursery

Because third-trimester ultrasounds are not routinely obtained in pregnancy, it is not uncommon for the first signs of achondroplasia to be recognized after delivery, in the newborn nursery, or at a first well-child visit. A pediatrician may notice during a newborn examination the presence of macrocephaly, disproportionate short limbs, rhizomelia, deviation between the third and fourth digits (so-called “trident hands”), and/or midface hypoplasia. In this case, x-rays of the spine and lower extremities and/or genetic testing can confirm the diagnosis. Once the diagnosis is confirmed, it is imperative that the patient be referred to a skeletal dysplasia team or a physician who can lead and coordinate skeletal dysplasia care for the infant. This care will entail screening and monitoring for the most common potential complications associated with achondroplasia in infancy, including the following:

### Foramen Magnum Stenosis

All infants with achondroplasia have a narrowed FM compared to the FM of an average-stature infant. However, in approximately 20% of infants with achondroplasia, there is critical FM stenosis, almost always seen before age 3 years, leading to compression of the neural and vascular structures at the base of the skull (29). FM stenosis leading to cervicomedullary compression is one of the known causes of unexpected death in infants with achondroplasia and likely the etiology of 12% mortality observed previously in children younger than 4 with achondroplasia (30). When cervicomedullary compression is severe, the stenosis may lead to central sleep apnea, neurologic compromise, and ultimately death. This is why aggressive early assessment to detect central sleep apnea and compression of the brainstem at the FM is necessary (1, 2). Monitoring for this serious condition includes an early sleep study, frequent neurologic examinations, and assessment of gross motor development over time. Polysomnogram is recommended within the first few months of life. FM stenosis is suspected and warrants neurosurgical consultation if there is a high central apnea index for age, hypoxia, and/or hypercarbia. A comprehensive neurologic examination at this age includes assessment of movement, tone, strength, cranial nerves, reflexes, and achondroplasia-specific developmental milestones as well as ability to feed. Frequent coughing or choking and subsequent suboptimal weight gain may be a bulbar sign that warrants further investigation. Evidence of hyperreflexia or clonus can be signs of spinal cord irritation and should lead to imaging. While cognition is not affected in achondroplasia, gross motor development is different in infants with achondroplasia with published expected timelines (1). Concern should arise if an infant's gross motor development is delayed compared to his or her achondroplasia peers. In addition to regular monitoring in the first few years of life, when available, obtaining a fast MRI of the brain and cervical spine can be a helpful screening tool. However, whenever concerns arise during an assessment, a full MRI of the cervical spine with flexion and extension views may be obtained (31). The decision to intervene with surgery should be based on a comprehensive assessment of growth, development, neurologic examination, imaging, and polysomnography.

### Hydrocephalus

Hydrocephalus is a less common complication of achondroplasia, with declining rates in the recent past due to changes in surgical technique. A recent study showed that 2.9% of infants with achondroplasia develop hydrocephalus that requires intervention (32). Monitoring for hydrocephalus includes obtaining regular head circumferences and ensuring they are plotted on an achondroplasia occipital frontal circumference growth curve. Teaching the family to assess the anterior fontanelle at regular intervals can be helpful (1).

### Apnea

Apnea can be observed in infants with achondroplasia for a number of reasons. As discussed above, central apnea can be a worrisome indication of critical FM stenosis. In addition, obstructive sleep apnea (OSA) is seen at a higher frequency in children with achondroplasia (33). This is typically due to a combination of narrowing of the midface structures and/or tonsillar/adenoid hypertrophy. OSA can also be seen at the infant stage, with some infants requiring respiratory support such as oxygen. Finally, care should be taken when infants with achondroplasia are placed in car seats. Macrocephaly and generalized hypotonia may cause the infant's head to fall forward, close the airway, and cause apnea. The goal is to position the infant in a car seat or car bed so as to mitigate the risk of accidental airway closure. A car seat test should be performed prior to discharge from the nursery, and a car bed may be needed for a short period of time until the infant gains better head control.

### Thoracolumbar Kyphosis

Infants with achondroplasia are born with a flexible thoracolumbar kyphosis (TLK). The family should be educated on the importance of protecting the spine by not propping the infant into head-over-spine sitting, maintaining reclined positioning, and ensuring the child is not placed in carriers, slings, or umbrella strollers that result in accentuation of the TLK.

### Growth and Development

Expectations around growth should be reviewed with the family, and it should be noted that, while average growth velocity is slower in children with achondroplasia, an infant will continue to grow in length and weight each day. Weight gain is expected to be slower than peers, at an average of 8 to 13 g/day in the first year of life (34). The child's total body length, weight, and head circumference should be plotted on achondroplasia-specific growth charts (1). Average stature body mass index growth charts are not an appropriate or useful tool; instead, using achondroplasia-specific weight-for-length or body mass index charts can be helpful in assessing proportions and growth over time (9, 10, 35). While speech and fine-motor milestones should be on par with peers, gross-motor milestone differences are expected in achondroplasia (1). There is a comprehensive developmental screening form available for children with achondroplasia (36). Gross-motor development is on an expected timeline for a child with achondroplasia. Infants who are meeting these expected milestones do not require engagement with physical therapy (1).

### Take Home Points for Case of Infant Presenting to Pediatric Endocrinologist

1. Every patient with achondroplasia deserves a health-care provider who will function as a coordinator of their health care, offering longitudinal surveillance, counseling, and treatment.
2. With recent approval of growth-modulating therapy for patients with achondroplasia, a pediatric endocrinologist may be the primary specialist for these patients.
3. There needs to be an individualized approach to counseling and discussion regarding medical management with realistic expectations. There is currently no evidence to date that vosoritide changes the rate of medical or orthopedic complications related to achondroplasia.

### Case 3: Infant Presenting to Pediatric Endocrinologist

A 3-week-old female infant was clinically diagnosed with achondroplasia by a pediatrician based on physical examination and a skeletal survey (x-rays). When researching treatment options, the infant's mother read that vosoritide was recently approved for newborns and wants to start medical treatment immediately. The pediatrician referred them to a geneticist for achondroplasia care; however, the first available visit was in 4 months in a different city 5 hours away. The infant's mother was reluctant to travel with her newborn, since she also read online that prolonged unattended sitting in a car seat can be dangerous for infants with achondroplasia. She decided to meet with the local pediatric endocrinologist. The pediatric endocrinologist completed a full physical examination and screened for concerning symptoms. The infant was growing well on achondroplasia-specific curves, neurological examination was appropriate, and development was on par for peers with achondroplasia. The physician discussed with the family the need for a baseline sleep study and an MRI to evaluate for FM stenosis prior to starting treatment with vosoritide. Referrals for an MRI of the brain and cervicomedullary junction, subsequent neurosurgery consultation, sleep medicine consultation with polysomnogram, and order for *FGFR3* genetic testing were placed. The mother inquires if they could start vosoritide as soon as treatment arrives. The pediatric endocrinologist educated the mother about the importance of surveillance, risk of infant death in achondroplasia, and provided a table created by the AAP on Health Supervision in Achondroplasia. The patient is scheduled with endocrine follow-up in 4 weeks and recommended the family try to schedule baseline MRI and sleep study as soon as possible.

## Discussion on Management by Pediatric Endocrinologist

Pediatric endocrinologists are experts in managing growth and genetic growth disorders; however, during their training, there is limited education and exposure to management of skeletal dysplasias (5). Emergence of growth hormone as an effective treatment for Turner syndrome and Prader-Willi syndrome led to the opportunity and need for pediatric endocrinologists to become experts in managing patients with these genetic conditions. Today pediatric endocrinologists are the most common specialist seeing these patients and others for growth hormone treatment (5). Prior to vosoritide, the medical home for a child with achondroplasia was rarely pediatric endocrinology; however, this paradigm may be shifting or shared with medical geneticists after approval of this growth medication.

For this 3-week-old whose mother is eager to start treatment, the pediatric endocrinologist needs to confirm the diagnosis, counsel the family, and manage expectations of medical treatment along with ensuring routine surveillance is performed. Since the pediatric endocrinologist will be writing the prescription for vosoritide, they may be the physician the family sees most often. The pediatric endocrinologist naturally becomes the medical home in this situation for the patient and family. Given that, it becomes their responsibility to monitor for potential complications related to achondroplasia. This is especially important in children younger than 5, as the risk of sudden death is 50 times higher than in the general population (2). Even though the pediatric endocrinologist may have limited experience, the importance of reviewing and following the surveillance guidelines by the AAP (1) and monitoring for these potentially life-threatening complications cannot be overstated.

In addition, the family needs to be counseled regarding these baseline risks so there are realistic expectations and understanding of the need to follow up with experts in a multidisciplinary medical team. The family needs to be educated on symptoms that should lead to medical attention. Finally, the endocrinologist should be up to date on the current data regarding vosoritide and share that information with the family. Currently vosoritide has only shown to increase height *z*-score from baseline (27).

During a discussion of management, the approach should be tailored to the family. For some, height may not be priority and the family may decide not to pursue treatment in infancy. The youngest patient started on vosoritide in the phase 2, double-blind, placebo-controlled study was aged 4 months (27). During counseling, it is important to recommend routine screening since there are no published data on the effect of starting vosoritide prior to age 4 months. Real-world data will emerge and needs to be published as more neonates with achondroplasia are started on treatment. Response to vosoritide in increasing height velocity has to also be assessed regularly in patients with achondroplasia despite having open growth plates since response is not the same in all patients with achondroplasia (8, 16). This has led to an increase in demand for improvement in expert guidelines for those starting vosoritide at birth until new evidence-based specific recommendations are published (16, 37, 38).

Family also needs to be educated on safely doing a long-distance drive with making sure infant is not sleeping alone in the car seat and to consider a car bed as they will need to drive long distances for seeing experts. For this infant who has limited access to a specialist, we need to understand barriers for seeking routine optimal care. It is estimated that 1 in 25 children miss a health-care appointment due to lack of transportation; these are more likely to be from low-income or rural areas (39). There are also barriers reported to access to a medical geneticist, pediatric orthopedics, and pediatric subspecialists due to insufficient number of providers, prolonged wait times, distances to seek care, lack of recognition among providers for the need of appropriate referrals, and inadequate insurance coverage (40-42).

### Take Home Points for Case of School-Age Child

1. Present all options regarding implementation of growth-modulating treatment (ie, starting before the child can assent, waiting until he or she can participate in the decision-making process, never starting treatment). For families proceeding with treatment, it is important that they be aware of the data and have realistic expectations based on accurate and up-to-date information.
2. The treatment landscape for children with achondroplasia is evolving. For current information about clinical trials, use clinicaltrials.gov.
3. Topics to address during a school-age clinic visit include language to discuss height with peers and adults in the school setting, classroom adaptations for equal access, and overall kindergarten preparedness.

### Case 4: School-Age Child

A 5-year-old girl is seen in a skeletal dysplasia clinic for a follow-up visit. She has a history of recurrent otitis media and conductive hearing loss that improved following myringotomy tube placement. She also has moderate obstructive sleep apnea for which she underwent a tonsillectomy and adenoidectomy. She is scheduled for a postoperative polysomnogram next month. The visit today is focused on readiness for kindergarten with an emphasis on accommodations that allow for age-appropriate independence in the classroom. On physical examination, she had a mild thoracolumbar kyphosis and genu varum, both of which are being monitored by the orthopedic surgeon on the multidisciplinary skeletal dysplasia team. Her height and weight for age are in the 15th percentile for girls with achondroplasia.

Her parents state they want to initiate vosoritide to help her grow and to decrease her risk of spinal stenosis in the future. The 5-year-old becomes tearful during the visit, expressing extreme distress about daily injections. The endocrinologist confirms vosoritide increased AGV by 1.57 cm/year during clinical trials, explaining that some children had a higher growth velocity increase while others had none. Furthermore, the endocrinologist explains that it is not known if orthopedic complications were altered by administration of vosoritide; this was not studied in the trial and may not be known for many years. Parents are reminded that they have the option *not* to treat their child at this time or ever; they can revisit this treatment option when their child is older and can participate in the decision-making process.

## Discussion on School-Age Child

Discussions around whether to pursue growth-modulating treatment are likely to occur at this visit; families need accurate data *before* the prescription is written. Vosoritide is the first growth modulator approved for achondroplasia in the United States. While growth hormone is a standard of care in other countries for achondroplasia, it is not used in the United States for this patient population due to lack of efficacy as well as increased risk of kyphoscoliosis development (1, 43). Vosoritide has been approved for children aged 5 and older since November 2021, when the pivotal trial showed increase AGV of 1.57 cm/year (44) and subsequent follow-up showed sustained growth in those responding. It is important to note that this is an average, and that some children do not demonstrate an increase in growth velocity. Thus far, there is no evidence that vosoritide changes the risk of developing orthopedic or medical complications related to achondroplasia, including FM stenosis or spinal stenosis. In addition to vosoritide, it is important to share with families that there are other medications that are currently in clinical trials and that the treatment landscape is evolving. Families interested in learning more can be referred to clinicaltrials.gov.

Approaching school age, there are many important medical and psychosocial topics to address. Most notably, accommodations in the school environment to allow the child to be as independent as his or her peers are essential. A 504 plan can be implemented to ensure modifications are in place throughout the school year. Accommodations may include step stools around the classroom, modifications to the desk and/or chair for back and leg support, and changes to the bathroom for accessibility. Some families may want to discuss how they are going to introduce dwarfism to a new school community and/or how to prevent bullying. This may be a time when the family chooses to provide education to teachers or the school regarding appropriate terminology (eg, *dwarfism*, *little person*, and *LP* are acceptable) vs terms/expressions that are considered offensive (eg, midget, height comparisons, and “normal” height instead of “average” are not appropriate). While there are no correct answers regarding these topics, the importance of parental awareness, open communication, and a de-emphasis on height for success cannot be overstated. Providing a child with the language to respond to their peers' questions is imperative and participation in a dwarfism social group can be incredibly empowering. In certain situations, child or family therapy can also be recommended.

By school age, a child with achondroplasia has likely outgrown some of the life-threatening concerns that existed in infancy. However, screening for other medical conditions is indicated, including OSA and chronic otitis media. Screening for OSA can include asking about snoring, feeling well-rested in the morning, existence of morning headaches, needing midday naps, and difficulty focusing. Chronic middle-ear fluid can present as frequent ear infections, difficulty hearing, speech delay, or articulation concerns. These issues should prompt further evaluation. In addition, at this age, a child with achondroplasia should be monitored by an orthopedic surgeon for genu varum and spinal stenosis, which may cause pain and alter their physical activities.

## Conclusion

In the setting of the approval of vosoritide, pediatric endocrinologists are gradually becoming a frequent medical home for children with achondroplasia. Given this, they need to ensure prior to starting or refilling prescriptions that they are following screening guidelines including adequate counseling, regular neurologic examinations, sleep apnea assessments, and imaging when needed. With the recent FDA approval of vosoritide for infants, there may be a need for expedited counseling and management, especially for those pursuing treatment in the first weeks or months of life. It is important to note that the achondroplasia medication landscape is rapidly changing; hence, this discussion should continue to be reassessed as more growth-modulating treatments become available.

## Data Availability

Data sharing is not applicable to this article as no data sets were generated or analyzed during the present study.

## Abbreviations

AAP: American Academy of Pediatrics
AGV: annual growth velocity
CNP: C-type natriuretic peptide
FDA: Food and Drug Administration
*FGFR3*: fibroblast growth factor receptor 3 gene
FM: foramen magnum
MRI: magnetic resonance imaging
NIPT: noninvasive prenatal testing
OSA: obstructive sleep apnea
TLK: thoracolumbar kyphosis
